# Prevascularization techniques for dental pulp regeneration: potential cell sources, intercellular communication and construction strategies

**DOI:** 10.3389/fbioe.2023.1186030

**Published:** 2023-05-18

**Authors:** Qiao Ruan, Shenglong Tan, Li Guo, Dandan Ma, Jun Wen

**Affiliations:** Department of Endodontics, Stomatological Hospital, School of Stomatology, Southern Medical University, Guangzhou, Guangdong, China

**Keywords:** prevascularization, dental pulp regeneration, tissue engineering, dental stem cells, endothelial cells, pericytes

## Abstract

One of the difficulties of pulp regeneration is the rapid vascularization of transplanted engineered tissue, which is crucial for the initial survival of the graft and subsequent pulp regeneration. At present, prevascularization techniques, as emerging techniques in the field of pulp regeneration, has been proposed to solve this challenge and have broad application prospects. In these techniques, endothelial cells and pericytes are cocultured to induce intercellular communication, and the cell coculture is then introduced into the customized artificial vascular bed or induced to self-assembly to simulate the interaction between cells and extracellular matrix, which would result in construction of a prevascularization system, preformation of a functional capillary network, and rapid reconstruction of a sufficient blood supply in engineered tissue after transplantation. However, prevascularization techniques for pulp regeneration remain in their infancy, and there remain unresolved problems regarding cell sources, intercellular communication and the construction of prevascularization systems. This review focuses on the recent advances in the application of prevascularization techniques for pulp regeneration, considers dental stem cells as a potential cell source of endothelial cells and pericytes, discusses strategies for their directional differentiation, sketches the mechanism of intercellular communication and the potential application of communication mediators, and summarizes construction strategies for prevascularized systems. We also provide novel ideas for the extensive application and follow-up development of prevascularization techniques for dental pulp regeneration.

## 1 Introduction

The loss of tooth vitality and subsequent complications caused by traditional root canal therapy are contrary to the concept of regeneration in modern stomatology. Pulp regeneration tissue engineering has become a new approach to solving this dilemma. The basic metabolism of the engineered tissue transplanted into the pulp chamber requires microvasculature to exchange nutrients and gases, remove metabolic waste and maintain a series of physiological functions. However, the diffusion limit of oxygen in the vasculature is only 200 μm (Hoeben et al., 2004). Therefore, the rapid vascularization of engineered tissue with a diameter larger than the critical value is the key to its survival, and the formation of a functional vascular network is the decisive factor in pulp tissue regeneration (Xie et al., 2021). In recent years, a large number of studies have used cell homing or stem cell transplantation to construct engineered tissue for *in situ* angiogenesis in the pulp chamber. By inducing the invasion of the host vasculature, the vascular network of engineered tissue was formed by the sprouting, extension and fusion of blood vessels to promote tissue regeneration. However, three points must be considered: 1) the host blood vessel could enter the pulp chamber only through a narrow apical foramen and grew along the root canal to the crown at a slow extension rate of 5 μm/h (Utzinger et al., 2015); 2) the pulp chamber constituted a naturally ischemic and hypoxic environment, which was not conducive to the survival of engineered tissue or to pulp regeneration; and 3) it was difficult to monitor and control the survival and vascularization process after tissue implantation. Therefore, researchers proposed prevascularization techniques in which a functional vascular network is preformed in engineered tissue constructs of dental pulp to ensure the rapid delivery of a sufficient blood supply. Subsequently, the vascular network anastomoses with the host vasculature (Ben-Shaul et al., 2019) and finally induces the regeneration of vascularized dental pulp (Mazio et al., 2019; Rademakers et al., 2019). Compared with traditional methods, this method could accurately control the formation of the vascular network, accelerate the process of vascularization *in vivo*, and facilitate large-scale production and clinical transformation.

To achieve the prevascularization of dental pulp, researchers obtain endothelial cells (ECs) and pericytes, introduce the cell coculture into the customized artificial vascular bed or induce self-assembly of the cell coculture to construct a prevascularization system, and then induce formation of a functional capillary network through intercellular communication and the interaction between cells and the extracellular matrix (cell‒ECM interaction). However, the cell sources and their quality of prevascularized dental pulp-engineered tissue are limited, the utilization of intercellular communication mechanisms is inadequate, and the construction strategies of prevascularization systems are still in their infancy. Therefore, this review considers dental stem cells as a potential cell source of endothelial cells and pericytes, discusses strategies for their directional differentiation, sketches the mechanism of intercellular communication and the potential application of communication mediators, summarizes construction strategies for prevascularized systems, and focuses on the recent advances in the application of prevascularization techniques for pulp regeneration.

## 2 Principles of vasculogenesis and angiogenesis

Prevascularization techniques preconstruct a functional vascular network through angiogenesis and vasculogenesis of ECs and perivascular cells (pericytes and vascular smooth muscle cells) (Liu et al., 2019). Vasculogenesis begins with the differentiation of the mesoderm into vascular cells and the formation of blood islands during embryonic development. The outer cells of the blood island are induced by vascular endothelial growth factor (VEGF) and fibroblast growth factor (FGF) to differentiate into ECs, while the inner region forms a lacuna in which the free cells differentiate into hematopoietic stem cells. Later, primitive blood vessels are formed (Carmeliet, 2000a). The maturation of primitive blood vessels is completed by the basement membrane and perivascular cells. Angiogenesis begins with the secretion of proangiogenic factors such as VEGF and FGF at injured or hypoxic sites (Draoui et al., 2017), forming a concentration gradient, activating the formation of endothelial tip cells, and directionally invading and decomposing the extracellular matrix (ECM). Tip cells and adjacent perivascular cells migrate along the concentration gradient of proangiogenic factors, followed by endothelial stem cells. These ECs proliferate to form new endothelial cell strips, then become lumen, and finally mature into functional vessels under the action of perivascular cells (Armulik et al., 2011; Wang et al., 2020).

The development of prevascularization techniques in the field of dental pulp regeneration originated from an attempt to construct blood vessels with endothelial cells (Athirasala et al., 2017). At present, this has progressed to the successful integration of ECs and pericytes and the formation of capillary networks (Qi et al., 2021). Few studies have investigated vascular smooth muscle cells. Therefore, this article mainly refers to ECs and pericytes. An in-depth study of vascular smooth muscle cells in dental pulp is likely to be a future development direction that has the potential to achieve the regeneration of microvasculature including capillaries, arterioles, and venules.

## 3 Potential cell sources of prevascularization techniques

### 3.1 Potential cell sources of ECs

ECs line the inner surface of blood vessels to form an inner wall, which is an indispensable part of the process of vascularization. Autologous ECs, such as human umbilical vein endothelial cells (HUVECs), human umbilical artery endothelial cells, human microvascular endothelial cells and endothelial progenitor cells, are the most direct sources of cells that can minimize the risk of immune rejection. These ECs are widely used in tissue engineering and vascular engineering for pulp regeneration. However, problems such as difficulty in obtaining samples, inefficient proliferation and expansion, rapid loss of expression of phenotypic markers *in vitro*, and potential dysfunction of ECs in some patients with systemic diseases have limited the application of autologous ECs (Kim and von Recum, 2008; Afra and Matin, 2020). With the rapid development of cell engineering and genetic engineering, the differentiation of stem cells, such as embryonic stem cells, induced pluripotent stem cells (iPSCs) and adult stem cells (ASCs), has been a promising method to obtain ECs.

Embryonic stem cells are not included in this discussion due to the destructive acquisition process and ethical limitations.

Pluripotent cells transformed from terminally differentiated cells by introducing specific transcription factors (Takahashi et al., 2007; Yu et al., 2007) or using small-molecule compounds (Guan et al., 2022) are called iPSCs. iPSCs have the potential to differentiate into all three germ lineages and have greater inherited plasticity than autologous ECs (Sivarapatna et al., 2015; Xu M. et al., 2019). However, the unlimited proliferative potential of iPSCs creates a high risk of teratoma or tumor formation. The process of producing iPSC-ECs is complex and time-consuming, and the phenotypes of cells that are recoded into a pluripotent state and then redifferentiated are usually immature (Yamanaka, 2020).

ASCs are undifferentiated cells that exist in differentiated organs and tissues. Among them, mesenchymal stem cells (MSCs) are multipotent stem cells derived from the mesoderm that were first successfully isolated from bone marrow (Friedenstein et al., 1976) and later obtained from adipose tissue (Zuk et al., 2001), teeth (dental stem cells) and other tissues. Among them, dental stem cells (DSCs), including dental pulp stem cells (DPSCs) (Gronthos et al., 2000), stem cells of human deciduous exfoliated teeth (SHEDs) (Miura et al., 2003), stem cells from apical papilla (SCAPs) (Sonoyama et al., 2006) and dental papilla cells, which are separated from permanent dental pulp, deciduous dental pulp, apical papilla tissue of immature young permanent teeth and dental papilla in tooth germ respectively, have the advantages of easy access and culture, high proliferative activity and the potential to differentiate into three germ layers while avoiding ethical and tumorigenic risks. Previous studies have verified that endothelial differentiated DPSCs promote the formation of prevascularized structures and the regeneration of vascularized pulp-like tissue (Katata et al., 2021). Therefore, the following section considers DSCs as a source of endothelial cells and reviews strategies for the endothelial differentiation of DSCs.

#### 3.1.1 Strategies for endothelial differentiation of DSCs

**FIGURE 1:**
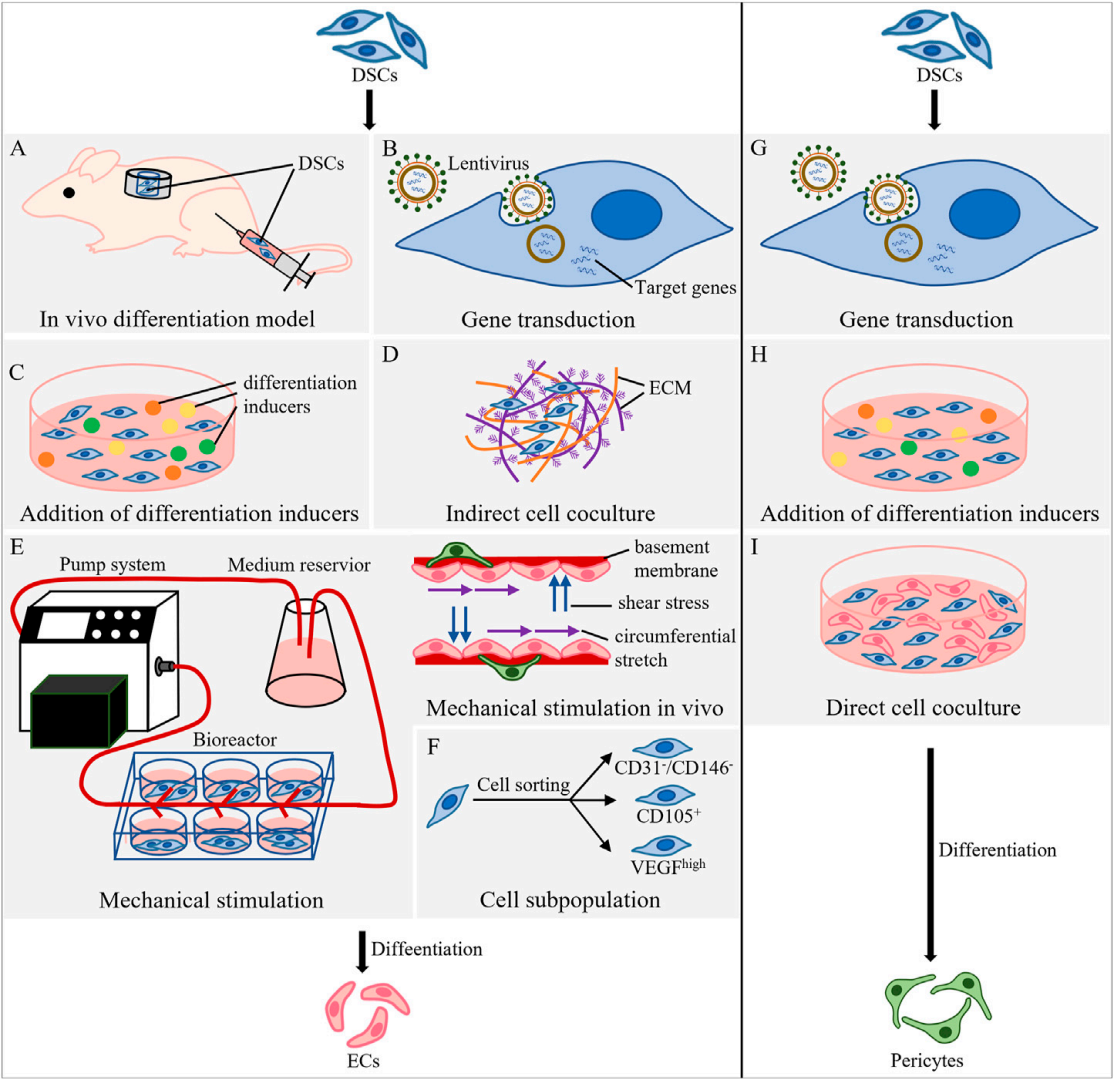
Methods of DSC differentiation into ECs and pericytes. **(A–F)** Strategies for endothelial differentiation of DSCs. **(A)** Root ectopia model and hindlimb ischemia model. In the root ectopia model, tooth slices loaded with DSCs are transplanted subcutaneously into nude mice; in the hindlimb ischemia model, DSCs are injected into the ischemic gastrocnemius muscle of the thigh. **(B)** Use lentivirus as a vector to integrate the target gene into DSCs and guide endothelial differentiation. **(C)** Addition of differentiation inducers to induce endothelial differentiation of DSCs. **(D)** Indirect contact coculture of DSCs and ECM of cells. **(E)** A pump system, medium reservoir and bioreactor were used to simulate mechanical stimulation *in vivo*. **(F)** Cell subpopulations are selected from DSCs. **(G–I)** Methods of DSC differentiation into pericytes. **(G)** Use lentivirus as a vector to integrate the target gene into the host and guide differentiation into pericytes. **(H)** Addition of differentiation inducers to induce DSCs to differentiate into pericytes. **(I)** Direct cell coculture of DSCs and ECs.

Existing studies mainly induce endothelial differentiation of DSCs through *in vivo* differentiation models or *in vitro* induction methods. The latter contains gene transduction, addition of differentiation inducers, cell coculture, and auxiliary strategies including mechanical stimulation and cell subgroup selection (Figure 1).

##### 3.1.1.1 *In vivo* differentiation model

*In vivo* differentiation models (Figure 1A) include the root ectopia model and the hindlimb ischemia model (Xu M. et al., 2019). In the root ectopia model, tooth slices loaded with labeled human DPSCs (hDPSCs) (Zhang et al., 2016) or SHEDs (Cordeiro et al., 2008) were transplanted subcutaneously into nude mice. The microvascular structure was observed in the newly formed pulp tissue, and the vascular wall was found to be lined with human hDPSCs or SHEDs differentiated into ECs (Sakai et al., 2010). The hindlimb ischemia model was established by ligating or cutting off the blood supply artery of the rat or mouse hindlimb, and then DSCs were injected into the ischemic gastrocnemius muscle of the thigh to obtain endothelial differentiation. Injection of DPSCs mobilized by granulocyte-colony stimulating factor into this model, which compared with colony-derived DPSCs, significantly increased capillary density and blood flow perfusion, and the area of regenerated pulp and the vascularized area formed in the heterotopic root model were significantly increased (Murakami et al., 2013).

Although *in vivo* models are closer to the human microenvironment, such models involve complex cellular and molecular cascade reactions, which often lead to failure due to local chronic inflammation (Xu M. et al., 2019). Therefore, *in vitro* induction methods have become the mainstream of endothelial differentiation of DSCs.

##### 3.1.1.2 *In vitro* induction method: gene transduction

Gene translation integrates target genes into the host to guide the expression of specific genes. Most studies apply lentivirus as a vector for the transfection target genes (Figure 1B). The ETS variant transcription factor 2 (*ETV2*) is the key gene for endothelial cell differentiation (Koyano-Nakagawa and Garry, 2017; Kim et al., 2023). Overexpression of *ETV2* was found to significantly enhance the endothelial differentiation of DPSCs (Li et al., 2021). *VEGF* regulates ECM remodeling and angiogenesis (Carmeliet, 2000b), while C-X-C motif chemokine ligand 12 (*CXCL12*) matures and stabilizes *VEGF*-induced neovascularization (Grunewald et al., 2006). Overexpression of *VEGF* and *CXCL12* in DPSCs resulted in a significant increase in the length and vascular area density of regenerated pulp-like tissue in the root canal (Zhu et al., 2019). Wnt ligand binds low-density lipoprotein receptor-associated protein 6 expressed by DPSCs to activate the Wnt/β-catenin pathway, inducing the endothelial differentiation of DPSCs. Therefore, forced activation of low-density lipoprotein receptor-associated protein 6 signaling may accelerate the generation of functional vessels (Silva et al., 2017). Hypoxia causes the stable expression of hypoxia inducible factor 1 subunit alpha (*HIF-1α*) and upregulation of *VEGF* (Pugh and Ratcliffe, 2003), promoting endothelial differentiation of SHEDs (Han Y. et al., 2022), while BCL2 apoptosis regulator (*BCL2*) is an anti-apoptotic gene that can inhibit cell apoptosis and prolong cell life (Vaux et al., 1988). Overexpression of *BCL2* and hypoxic preconditioning could enhance cell survival and angiogenesis after DPSC implantation (Dissanayaka et al., 2020). In addition, some microRNAs are involved in regulating the angiogenesis of human dental pulp cells (hDPCs). Knocking down the expression of miR-424 in hDPCs could promote endothelial differentiation and tube formation (Liu et al., 2014).

##### 3.1.1.3 *In vitro* induction method: addition of differentiation inducers

The addition of differentiation inducers, including growth factors and signaling pathway regulators (Le Bras et al., 2010), can induce endothelial differentiation of DSCs (Figure 1C), and this process which can be further enhanced by epigenetic regulators and epigenetic regulatory factors.

Efficient endothelial differentiation and angiogenesis of DPSCs has been achieved in serum-free medium supplemented with vascular growth factors VEGF-A165, B27 and heparin (Luzuriaga et al., 2020). Commercially available endothelial cell growth medium EGM-2™ similar to this formula achieve the same goal (Aksel and Huang, 2017). Endothelial differentiation medium supplemented with 50 ng/mL recombinant human VEGF165 induces three-dimensional (3D) DPSC constructs to form endothelial differentiation cell constructs. On the 10th day, the outer layer of endothelial differentiation cell constructs has formed a vascular network composed of VWF-positive cells. Pulp-like tissue with a high density of CD31^+^ blood vessels will ultimately form in the root ectopia model (Katata et al., 2021). Under hypoxic conditions, the kidney produces erythropoietin, whose angiogenic potential is close to that of some other known angiogenic factors, such as VEGF and basic fibroblast growth factor (FGF2). Therefore, short-term exposure to the combination of serum deprivation, glucose deprivation and hypoxia/oxygen deprivation promoted the differentiation of SCAPs into endothelial cells; the addition of recombinant human erythropoietin to complete culture medium-α, SCAPs formed a microvascular structure, and the upregulation of vascular endothelial growth factor receptor 2 (VEGFR2), von Willebrand factor (VWF), vascular endothelial cadherin (VE-cadherin) and other endothelial markers was observed (Koutsoumparis et al., 2018).

Semaphorin-4D is a transmembrane class IV semaphorin that participates in the regulation of nerve signal transduction, immunity, angiogenesis and other processes (Zhang L. et al., 2022). By binding with plexin B1 on the cell membrane of DPSCs, Semaphorin-4D activates the PI3K/AKT and MEK/ERK1/2 signaling pathways, leading to the upregulation of endothelial differentiation-related genes (*VEGFR1, VEGFR2, CD31* and *VWF*) and angiogenesis-related genes (*HIF,* angiopoietin 1 and angiopoietin like 4) and thereby inducing the endothelial differentiation of DPSCs (Zou et al., 2019). Transforming growth factor beta (TGF-β) inhibitor enhances the differentiation of dental pulp-derived stem cells into endodermal cells (Xu et al., 2018). This inhibitor acts on the TGF signaling pathway (TGFβ/Smad2/3), inhibits the transformation of SHEDs into smooth muscle cells (SMCs), and promotes endothelial differentiation of SHEDs through the VEGF signaling pathway. VEGF triggers MEK1/ERK phosphorylation and downstream signaling pathway activation and participates in SHED endothelial differentiation (Bento et al., 2013) and the sprouting and anastomosis of vessels derived from DPSCs (Sasaki et al., 2020). Therefore, the MEK1/ERK activator may contribute to the endothelial differentiation of DPSCs. In addition, a glycogen synthase kinase 3 β (GSK-3b) inhibitor activated the canonical Wnt/β-catenin pathway to induce the endothelial differentiation of DPSCs (Zhang et al., 2016).

VPA is the epigenetic regulator most widely used in chemical reprogramming to overcome the epigenetic barriers between different types of cells. The combination of VPA with signal pathway regulators (VPA, GSK signal inhibitor, TGF-β signal inhibitor, cAMP signal activator and ROCK inhibitor) and three key growth factors (VEGF, BMP-4 and 8-Br-cAMP) effectively induced SCAPs into EC-like cells that formed vascular structures *in vivo* and *in vitro* (Yi et al., 2021). In addition, p53 is a tumor suppressor that not only induces cell apoptosis but also balances cell stemness and differentiation through p21. Bmi-1 is a polycomb protein that is involved in regulating stem cell differentiation as an epigenetic regulatory factor. p53 inhibitors can induce high expression of Bmi-1 and promote the endothelial differentiation of DPSCs by blocking the p53/p21 signaling pathway (Zhang Z. et al., 2021).

##### 3.1.1.4 *In vitro* induction method: cell coculture

The main methods of cell coculture are direct contact and indirect contact coculture (Figure 1D). The study of endothelial differentiation of DSCs by direct contact culture is still lacking. In indirect coculture, the vascular tissue with multiple cells or ECM of ECs provides DSCs with a microenvironment containing endothelial differentiation guidance clues consisting of a complex combination of proteins, polysaccharides, growth factors and cytokines (Xu M. et al., 2019). Bovine acellular dental pulp matrix retains the natural vascular system and angiogenic factors. The inoculation of hDPSCs into this matrix could upregulate the expression of hDPSC angiogenesis markers and cause vascular system repopulation (Alghutaimel et al., 2021). Moreover, after 7 days of coculture of acellular ECM from SHEDs and HUVECs, significant upregulation of the specific endothelial markers CD31 and VEGFR2 and the formation of more capillary structures on the matrix gel were observed (Gong et al., 2017). Furthermore, heparan sulfate in the ECM plays a key role in the endothelial differentiation of DPSCs (Li et al., 2023).

Among the above methods, chemical stimulation is the most common induction method with the highest efficiency, convenient operation, fine adjustment and low cost. However, the chemical stimulation method based on two-dimensional monolayer cell culture has difficulty imitating intercellular communication and cell‒ECM interactions in the microenvironment. Therefore, some scholars have chosen to apply natural ECM after acellularization treatment. However, decellularization techniques cannot balance the complete removal of cells and the maximum retention of ECM components (Gong et al., 2017). Other researchers have chosen a 3D culture that forms a 3D structure through the self-assembly of SHEDs (Guo et al., 2020) and DPSCs (Katata et al., 2021) or have prepared 3D tooth germ organ conditioned medium (Zhou et al., 2021), which is conducive to the endothelial differentiation of SHEDs and DPSCs and the formation of vascularized pulp tissue. However, 3D cultivation faces the risks of complex operation, high cost and low output. These methods need to be further developed.

##### 3.1.1.5 Auxiliary method for *in vitro* induction: mechanical stimulation

The life activities of cells occur in a dynamic, balanced microenvironment. Therefore, static culture conditions without mechanical stimulation, such as the hardness of the vascular intima or shear stress of blood flow, are not conducive to endothelial differentiation. ECs and pericytes jointly secrete ECM to form the basement membrane, which is composed of a protein fiber soft matrix with a low compression modulus (Peloquin et al., 2011). VEGF alone induced the expression of the early endothelial markers VEGFR1 and smooth muscle α-actin (SMA) in MSCs, which induced the MSCs to become vascular progenitor cells or enter a pathological vascular phenotype. The addition of soft matrix elasticity (∼2 kPa) could inhibit SMA and promote the expression of the mature endothelial cell markers VWF and VEGFR2 (Wingate et al., 2014). Therefore, the combination of mechanical and chemical stimulation accelerated the differentiation of MSCs into a mature vascular phenotype. In addition, SHEDs need physiological shear stress to activate angiogenic factors, initiate the VEGF-DLL4/Notch-ephrin B2 cascade, promote SHED differentiation into ECs (Wang et al., 2018) and maintain the function of vascular-related cells (Sharmin et al., 2021). Furthermore, the combination of fluid shear stress and cyclic stress upregulates the expression of EC markers in MSCs (Kim D. H. et al., 2016).

There are few literature reports relevant to dynamic cell culture conditions in dentistry, so we can learn from customized bioreactors in other fields. The cell culture medium inside this instrument was supplied by a fluid reservoir and perfused by a digital permanent pump to simulate an *in vivo* environment containing stimulatory effects such as fluid shear stress (Hann et al., 2021) (Figure 1E).

##### 3.1.1.6 Auxiliary method for *in vitro* induction: cell subpopulation

Sorting dental stem cell subpopulations with high vascular potential contributed to improving the efficiency of endothelial differentiation (Figure 1F). CD31^-^/CD146^-^ side population and CD105^+^ cells in the dental pulp group promoted an increase in capillary density and blood flow in a mouse hindlimb ischemia model (Nakashima et al., 2009). After 2 years, collagen scaffolds loaded with this subgroup of cells and CXCL12 were transplanted into the root canal, achieving the first complete *in situ* dental pulp regeneration full of blood vessels and nerves (Nakashima and Iohara, 2011). In cell subpopulation with VEGFR1^high^ SHEDs or DPSCs, VEGF signaling activates MEK1/ERK signaling through VEGFR1, inhibits STAT3 transcriptional activity, and differentiates DPSCs into ECs (Bergamo et al., 2021). A group of CD24a^+^ multipotent cells isolated from dental papilla cells by 3D sphere culture had stronger stemness and differentiation potential than other DSCs, and can be used as a cell source for the efficient regeneration of vascularized dental pulp (Chen et al., 2020).

### 3.2 Potential cell sources of pericytes

Pericytes cover the outside of the microvascular walls and are widely distributed in all tissues and organs of the body, regulating the sprouting of ECs, enhancing the formation of the EC barrier, and promoting the stability and maturation of capillaries (Armulik et al., 2011). Primary pericytes can be obtained by microvascular isolation and culture (Crisan et al., 2008). However, similar defects to those of autologous ECs limit the mass production of pericytes. Therefore, the identification of alternative sources of pericytes is helpful for experimental research and clinical transformation.

Studies have found that pericytes express surface markers of MSCs, namely, CD146^+^, CD45^−^, CD34^−^, CD73^+^, CD44^+^ and CD105^+^ (Crisan et al., 2008; Wang et al., 2019; Manocha et al., 2022), and exhibit multidirectional differentiation ability similar to that of MSCs (Farrington-Rock et al., 2004; Tsang et al., 2013; Chou et al., 2020; Kesavan et al., 2023). The MSCs residing in tissue are located in the pericyte niche in the microvasculature (Murray et al., 2014) and express perivascular cell markers: CD146, desmin, NG2, and platelet-derived growth factor receptor (PDGFR) (Crisan et al., 2008). Therefore, MSCs could be considered a source of pericytes for prevascularized tissue. In addition, pericytes are tissue specific (Yianni and Sharpe, 2018), and MSCs derived from the same tissue are more conducive to revascularization (Meijer et al., 2022). Therefore, DSCs are preferred as the periodontal cell pool for dental pulp engineering tissue (Figure 1).

#### 3.2.1 Methods for DSC differentiation into pericytes

The strategies for acquiring pericytes included gene transduction (Figure 1G), chemical stimulation (Figure 1H) and cell coculture (Figure 1I). DPSCs transfected with hypoxia-responsive elements and *FGF2* differentiated into CD13^+^ pericytes and secreted FGF2 to improve the expression of N-cadherin and promote the adhesion of CD13^+^ pericytes to endothelial cells (Zhu S. et al., 2021). NG2^+^ hDPCs sorted from hDPCs were cultured in human pericyte medium and differentiated into NG2^+^ pericytes (Fu et al., 2023). In addition, FGF2 in endothelial cell growth medium induced DPSCs to differentiate into NG2^+^ pericytes and further supported the proliferation and migration of pericytes (Delle Monache et al., 2019).

Cell coculture is not only a strategy to obtain pericytes but also a method to identify the function of pericytes. Coculture with HUVECs verified the pericyte function of SHEDs and DPSCs (Zhu S. Y. et al., 2021). Among them, SHEDs and HUVECs separately expressed angiogenesis-related factors and their corresponding receptors: SHEDs expressed VEGF, CXCL12, and platelet-derived growth factor receptor (PDGFRB), while HUVECs expressed VEGFR1, VEGFR2, C-X-C motif chemokine receptor 4 (CXCR4), and platelet-derived growth factor BB (PDGF-BB), mediating the interaction between pericytes and ECs (Kim J. H. et al., 2016). DPSCs mainly play a VEGFR2-dependent pericyte function when cocultured with HUVECs or primary endothelial cells isolated from the hearts of mice (Janebodin et al., 2013). In addition, Aksel induced human DPSCs to differentiate into ECs in endothelial growth medium and cocultured them with noninduced DPSCs to form a vascular network. As a result, noninduced DPSCs play the role of pericytes and maintain the stability and integrity of blood vessels (Aksel and Huang, 2017). In fact, DSC-EC coculture is the mainstream technique for pulp prevascularization at present. Intercellular communication between DSCs and ECs could effectively induce ECs and DSCs to perform angiogenesis and act as pericytes, respectively, and further form vascularized engineered tissue.

## 4 Intercellular communication in prevascularization techniques

The processes of vasculogenesis and angiogenesis in prevascularization techniques are complex and precise, and are induced via intercellular communication between ECs and pericytes (Armulik et al., 2005). This intercellular communication involves paracrine and juxtacrine signal transduction (Table 1). Paracrine signaling mediators are mainly bioactive molecules, so artificial addition of angiogenic bioactive molecules can improve the efficiency of revascularization of engineered tissue. Ratajczak’s review has fully revealed the angiogenic factors in DSCs, which can be referred to in the table in this paper (Ratajczak et al., 2016). Furthermore, the natural bioactive molecular pool taken from organisms provides a large number of angiogenic factors on the basis of preserving the microenvironment *in vivo*. Rapid progress has been made in laboratory research and clinical transformation (Table 2).

**TABLE 1:** Intercellular communication in dental pulp prevascularization.

Modes	Donor cells	Mediators	Receptors on recipient cells	Signaling pathways	Roles	References
Paracrine; autocrine	Pericytes (DPSCs)	VEGF	VEGFR2 on ECs or pericytes (DPSCs)	VEGFR2/PLC/PIP2 (ECs); VEGFR2/Ras/Raf/MAPK (ECs); VEGFR2/PI3K/AKT (ECs); VEGFR2/ERK1/2 (DPSCs)	Enhancing the invasion, proliferation, migration, survival, permeability, and activation of ECs; enhancing the VEGF signal in pericytes (DPSCs)	Wong and Jin (2005), Grando Mattuella et al. (2007), Janebodin et al. (2013), Yuan et al. (2015), Delle Monache et al. (2019), Bergamo et al. (2021)
Paracrine	ECs (DPSCs)	PDGF-BB	PDGFRβ on pericytes (SHEDs) or SMC	PDGFR-β/PI3K/AKT	Recruiting SHEDs (to obtain a pericyte phenotype) or SMCs, and then maturing blood vessels	Atlas et al. (2021), Zhang et al. (2022a)
Paracrine	Both ECs and pericytes	TGF-β	ALK-1 on ECs	TGF-β/ALK1/Smad1/5	Enhancing the proliferation and migration of ECs and the formation of new blood vessels	Goumans et al. (2002), Kritharis et al. (2018)

Modes	Donor cells	Mediators	Receptors on recipient cells	Signaling pathways	Roles	References
Paracrine	ECs or pericytes (SHEDs)	TGF-β	ALK5 on pericytes (SHEDs) or ECs	TGF-β/ALK-5/Smad2/3	Inducing SHEDs to differentiate into SMCs, inhibiting the proliferation and migration of ECs, and promoting ECM production and vascular maturation	Goumans et al. (2002), Goumans et al. (2003), Van Geest et al. (2010), Xu et al. (2018)
Paracrine	Pericytes (DPSCs)	ANGPT1	TIE2 on ECs	ANGPT1/TIE2	Enhancing EC adhesion and inhibiting endothelial sprouting to enhance intercellular and vascular stability	Zhang et al. (2021a)
Juxtacrine	Pericytes (DPSCs, SCAPs)	EFNB2	EPHB4 on ECs	EFNB2/EPHB4	Inducing EC sprouting and lumen formation while inhibiting EC migration to mature blood vessels	Yuan et al. (2016), Gong et al. (2019), Yuan et al. (2019)

**TABLE 2:** Research status of potential signaling mediators in pulp revascularization.

Mediators	Results from *in vivo* or *in vitro* test	References
Adipose tissue-derived microvessel fragments	Dental pulp-like tissues formation with vascular networks *in vivo*	Xu et al. (2021)
Concentrated growth factor	Pulp-like tissues formation with angiogenesis and neurogenesis *in vivo*	Xu et al. (2019a)
Platelet lysate	Microvascular formation and pulp-like tissue regeneration *in vivo*	Zhang et al. (2021a)
Conditioned medium from DPSCs	Tubular network formation *in vitro*	Gharaei et al. (2018)
Conditioned medium from SHEDs	Vascularized pulp-like tissue formation *in vivo*	de Cara et al. (2019)
Extracellular vesicles derived from DPSCs	Tubular network formation *in vitro*	Zhang et al. (2020)
Extracellular vesicles derived from DPSCs pretreated with lipopolysaccharide	Pulp regeneration with new blood vessels *in vivo*	Chen et al. (2021)
Extracellular vesicles derived from Schwann cells	Dentin-pulp complex-like tissue formation containing newly formed vessels and nerve fibers *in vivo*	Wang et al. (2022)
Exosomes isolated from SHEDs	Angiogenesis and pulp tissue regeneration *in vivo*	Wu et al. (2021)
Exosomes derived from SHEDs pretreated with hypoxia	Tube formation *in vitro* and lumenal structures *in vivo*	Liu et al. (2022)
Apoptosis bodies isolated from SHEDs	Vascularized dental pulp tissue formation *in vivo*	Li et al. (2022)

In recent years, in-depth research on the paracrine substances of cells has revealed that in addition to common biologically active molecules, there are also extracellular vesicles (Table 2), which have been widely applied in regenerative medicine and vascular engineering (Hade et al., 2021; Huang et al., 2021). Although extracellular vesicles have not been used in the prevascularization of dental pulp engineered tissue, they have broad application prospects in the future.

### 4.1 Paracrine and juxtacrine pathways

The interaction between pericytes derived from DSCs and ECs involves paracrine and juxtacrine signal transduction. Paracrine signals are soluble molecules released to act on receptor cells in the microenvironment, and juxtacrine signals are transmitted through direct contact between adjacent cells. The following sections introduce VEGF/VEGFR, PDGF-BB/PDGFRB, TGFβ, angiopoietin-1/TEK receptor tyrosine kinase (ANGPT1/TIE2) and ephrin B2/EPH receptor B4 (EFNB2/EPHB4) (Table 1) (Gaengel et al., 2009).

VEGF is the most critical growth factor in vasculogenesis and angiogenesis and activates the PKC, MAPK, AKT and Ca2^+^-calcineurin signaling pathways. Endothelial differentiation of DPSCs is caused by the binding of VEGF and VEGFR1 (Bergamo et al., 2021), while the function of DPSCs as pericytes is VEGFR2 dependent, participating in tube formation of ECs in the early stage of EC angiogenesis (Yuan et al., 2015; Delle Monache et al., 2019). VEGF secreted by DPSCs acts on VEGFR2 on ECs via the paracrine pathway, activating the PLC/PIP2 (Wong and Jin, 2005), RAS/RAF/MAPK and PI3K/AKT signaling pathways and inducing the promotion, permeability, invasion, migration, survival and activation of ECs. At the same time, VEGF acts on VEGFR2 on DPSCs in an autocrine manner (Grando Mattuella et al., 2007), further enhancing the VEGF signal (Janebodin et al., 2013). In addition, few studies have investigated the VEGFR2/Calcineurin/NFAT signaling pathway in the field of pulp prevascularization and regeneration, although this pathway has been confirmed to be involved in tumor angiogenesis and vasculogenesis (Alghanem et al., 2017). The activity of NFAT target genes is closely related to the activation of ECs (Suehiro et al., 2014).

ECs release PDGF-BB, which combines with PDGFRB on the surface of pericytes. Then, PDGFR-positive pericytes are recruited to cover the vascular wall. When cocultured with human umbilical artery smooth muscle cells, human DPSCs differentiated into ECs and released PDGF-BB to recruit SMCs. Inhibition of PDGFRB signaling reduced the number of vascular buds and the recruitment of SMCs around the vessels (Zhang Z. et al., 2022). When cocultured with HUVECs, SHEDs were recruited by PDGF-BB released by HUVECs to the periphery of blood vessels to obtain a pericyte phenotype and deposit type IV collagen on the basement membrane. This coculture could effectively prevascularize constructs and mature microvessels, and improve blood perfusion and cell survival after transplantation (Atlas et al., 2021).

Both ECs and pericytes express TGF-β and TGF-β receptors; therefore, the TGF-β signaling pathway is related to the proliferation and differentiation of ECs and pericytes. TGF-β receptors, including type I TGF-β receptor activin receptor-like kinase-1 (ALK1) and activin receptor-like kinase-5 (ALK5), trigger different cellular effects (Goumans et al., 2002; Goumans et al., 2003). In most cell types, TGF-β activates ALK5, causes Smad2/3 phosphorylation (Van Geest et al., 2010), induces MSCs to differentiate into SMCs (Xu et al., 2017), inhibits the proliferation and migration of ECs, and promotes ECM production and vascular maturation. The TGF-β inhibitors used in a previous article inhibited this pathway and induced the endothelial differentiation of DPSCs (Xu et al., 2018). Only ECs expressed ALK1, and the activation of ALK1 caused Smad1/5 phosphorylation, promoted the proliferation and migration of ECs, and led to the formation of new blood vessels (Goumans et al., 2002; Kritharis et al., 2018).

Perivascular cells, including perivascular MSCs, express ANGPT1 to recruit and maintain supporting cells around endothelial cells (Kim et al., 2000) and promote vascular maturation by strengthening the interaction between perivascular cells and ECs (Davis et al., 1996). ANGPT1 binds with the receptor TIE2 on the surface of the EC membrane and participates in the regulation of vascular stability. In a previous study, DPSCs treated with TGF-β (T-DPSCs) triggered the phosphorylation of Smad2/3. Activated Smad2/3 further induced the upregulation of ANGPT1 and VEGF gene expression. First, the binding of VEGF secreted by T-DPSCs and VEGFR2 on ECs induces vascular germination. Subsequently, ANGPT1 secreted by T-DPSCs activated the receptor TIE2 on the surface of the HUVEC membrane. Activated ANGPT1/TIE2 increased the expression of VE-cadherin, enhanced intercellular adhesion, and inhibited the migration of ECs and endothelial sprouting, thus stabilizing blood vessels. Meanwhile, T-DPSCs differentiated into pericyte-like cells, so the VEGF secreted by DPSCs gradually decreased, which weakened the VEGF/VEGFR2 signal, inhibited HUVEC migration, and promoted vascular maturation in cooperation with ANGPT1/TIE2 (Zhang Y. et al., 2021).

In addition, the EFNB2/EPHB4 system is a contact-dependent bidirectional juxtacrine signaling pathway that regulates embryonic vascular development and postnatal angiogenesis (Salvucci and Tosato, 2012). Gong first revealed that the EFNB2/EPHB4 signaling pathway played a key role in the regulation of EC sprouting and lumen formation by DPSCs (Gong et al., 2019), while Yuan found that EPHB2 stabilized the vascular structure produced by HUVECs and SCAPs (Yuan et al., 2016). SCAPs overexpressing EPHB2 secreted more VEGF and accelerated angiogenesis by binding with VEGFR2 on HUVECs. Simultaneously, activation of the EPHB2 reversed signal in SCAPs might activate the ANGPT1/TIE2 axis in HUVECs and regulate vascular permeability and morphogenesis (Yuan et al., 2019).

Through an understanding of the mechanism of EC-pericyte intercellular communication, we can consider the incorporation of key signal molecules or signal pathway regulators to accelerate the process of prevascularization of dental pulp constructs, which requires further research.

### 4.2 Natural signal molecular storage

Adipose tissue-derived microvessel fragments are functional vascular fragments isolated from adipose tissue and contain angiogenic factors such as VEGF and FGF, as well as cell components such as ECs and pericytes, which are required for vascularization. Aggregates obtained by the coculture of these microvessel fragments and DPSCs have been found to promote the vascularization of regenerated pulp (Xu et al., 2021).

There are many kinds of platelet derivatives. The concentrated growth factor obtained by the differential centrifugation of human venous blood is a third-generation platelet concentrate containing a large number of angiogenesis-related factors, including PDGF-BB, TGF-β1, insulin like growth factor 1, VEGF and FGF2 in natural fibrin network structures were considered likely to serve as signal molecule reservoirs and scaffolds for pulp prevascularization. After implanting concentrated growth factor into the root canal of immature permanent teeth of beagle dogs, pulp-like tissues that resembled the natural tissues and exhibited vascularization and innervation were regenerated after 8 weeks (Xu F. et al., 2019). Platelet lysate (PL) is obtained by repeatedly freezing and thawing platelets or by ultrasound and contains a high concentration of bioactive molecules. Many studies have incorporated PL into bioactive scaffold materials to encapsulate HUVECs and DPSCs (detailed in the next chapter) to accelerate the process of vascularization in engineered tissues.

MSCs secrete a great quantity of paracrine factors into the medium during the culture process. Such conditioned medium has been proven to contribute to tissue regeneration. Conditioned medium from DPSCs promoted the adhesion, proliferation, migration and tubular network formation of ECs (Gharaei et al., 2018). Based on this study, conditioned medium from SHEDs induced the formation of vascularized pulp-like tissue in the root canal of rats (de Cara et al., 2019).

### 4.3 Potential applications of extracellular vesicles

Extracellular vesicles (EVs) are membranous vesicles released from cells (van Niel et al., 2018) with subgroups that include exosomes, apoptotic bodies, and microvesicles, all containing a large number of nucleic acids and proteins. Zhang fabricated an EV-fibrin gel composite in which EVs were isolated from DPSCs and then mixed with fibrin gel. Thereafter, ECs and DPSCs were cocultured in EV-fibrin gels. DPSC-EVs promoted the rapid formation of vascular structures by increasing the release of VEGF (Zhang et al., 2020). hDPSCs pretreated with lipopolysaccharide (in a mild inflammatory environment) could increase EV production and further enhance angiogenic potential, possibly through TGF-β signaling (Chen et al., 2021). Schwann cells are glial cells in dental pulp tissue, and their extracellular vesicles enhance the recruitment of ECs and MSCs and introduce the formation of dental pulp-like tissue in *in vivo* models (Wang et al., 2022).

Exosomes are secreted by the fusion of multivesicular bodies and the plasma membrane (Han Q. F. et al., 2022). MiR-26a secreted by exosomes derived from SHEDs (SHED-Exos) regulates the TGF-β/SMAD2/3 signaling pathway to improve angiogenesis in SHEDs and HUVECs, contributing to pulp tissue regeneration (Wu et al., 2021). Compared with SHED-Exos, SHED-Exos pretreated with hypoxia further promote the proliferation, migration and tubular formation of ECs and contribute to increasing the expression of VEGF and the number of CD31^+^ lumenal structures by transferring let-7f-5p and miR-210-3p (Liu et al., 2022). Exosomes isolated from DPSCs cultured under a growth or angiogenic differentiation condition contain key miRNAs for angiogenesis (miR-199a-3p, miR-221-3p, miR-24-3p, miR-21-5p). The miRNA sequences loaded in exosomes have significant therapeutic potential for pulp prevascularization (Ganesh et al., 2022).

Apoptosis bodies are produced during cell apoptosis, by being released from SHEDs. These bodies carried mitochondrial Tu translation elongation factor, regulate the angiogenic activation of ECs via the transcription factor EB-autophagy pathway, and promote the formation of vascularized dental pulp tissue (Li et al., 2022).

## 5 Construction strategies for prevascularization systems

**FIGURE 2:**
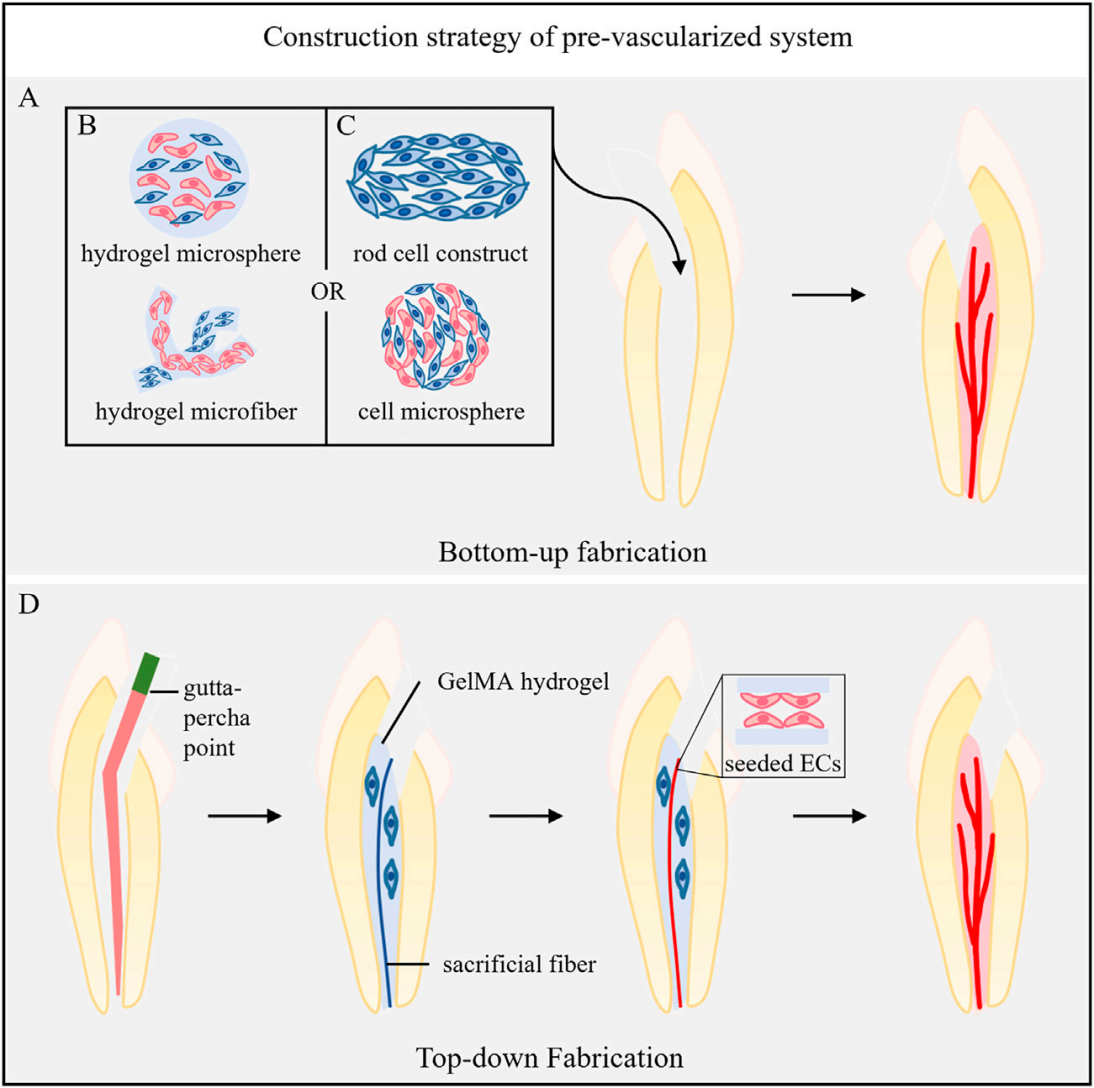
Construction strategy of the prevascularized system. **(A)** Bottom-up fabrication. **(B)** The incorporation of ECs and pericytes into the materials. **(C)** Cultivation of vascular-related cells into scaffold-free, self-assembled 3D microtissue structures. **(D)** Top-down Fabrication. The gutta-percha points into the root canal to form tapered templates, and the hydrogel loaded with cells around it is injected. Then, gutta-percha points were removed after hydrogel coagulation, and sacrificial agarose material was injected. Finally, the sacrificial material is removed, and ECs are seeded in the formed microchannel.

Construction strategies for prevascularization systems are mainly divided into two types (Figure 2): 1) bottom-up strategies, in which the microenvironment in an organism is simulated to induce the formation of a self-assembled vascular structure (Figure 2A), and 2) top-down strategies, in which a vascular bed with specific geometry and structure is designed and prepared in advance before introducing cells (Figure 2D).

### 5.1 Bottom-up fabrication

Bottom-up fabrication includes two methods: 1) the incorporation of ECs and pericytes into the biological microenvironment simulated by the materials (Figure 2B) or 2) the cultivation of vascular-related cells into scaffold-free and self-assembled 3D microtissue structures (Figure 2C). Both methods aim to induce vasculogenesis and angiogenesis and eventually form microvascular networks.

#### 5.1.1 Scaffold system made from natural materials or synthetic materials

Each step in the process of vascularization is regulated by microenvironmental clues provided by the surrounding ECM (cell‒ECM interaction). Therefore, the type of scaffold material and its physicochemical properties and biological characteristics affect the formation of the vascular system of the engineered tissue (Marchand et al., 2019). During angiogenesis, fibronectin, laminin, collagen, hyaluronic acid and other proteins or polysaccharides in the ECM participate in regulating the proliferation, differentiation and sprouting process of ECs (Shafiee et al., 2021) Natural hydrogel materials, such as collagen, gelatin, alginate and hyaluronic acid, are rich in the above ingredients and have a strong ability to promote angiogenesis (Shokrani et al., 2022). Their high water content and porosity can simulate the natural ECM (Klotz et al., 2019). In previous studies, 3D collagen hydrogel and 3D fibrin gel were separately used as scaffolds for coculturing ECs and pericytes to achieve prevascularization of the construct (Gong et al., 2019; Atlas et al., 2021). Synthetic materials could be produced by the artificial modification of natural materials or completely synthesized to obtain customized properties suitable for the formation of vascular networks. Therefore, synthetic materials can break through the limitations of natural materials and are more favored by researchers.

##### 5.1.1.1 Natural scaffold based on chitosan

Chitosan hydrogel is a natural linear polysaccharide. In addition to its antibacterial properties, its application in hydrogel carriers can promote cell adhesion and migration. The author loaded the SHED secretome (conditioned medium from SHEDs) into chitosan hydrogel, which significantly increased the release of VEGF and CXCL12, and contributed to rapidly restoring the blood perfusion of dental pulp engineered tissues. Subsequently, the formation of controlled and sustained vascular structures was observed in the tube formation experiment when SCAPs were cocultured with HUVECs (Carvalho et al., 2022).

##### 5.1.1.2 Natural scaffold based on self-assembled peptide

The peptide components of the self-assembled peptide hydrogel PuraMatrix ™ self-assembled into a nanofiber network, creating a real 3D environment for cells. Its injectability was conducive to accurately matching the 3D shape of the dental root canal. Prevascularized microtissues formed by encapsulated DPSCs/HUVECs were transplanted into the root heterotopic model. Then, the formation of vascular structure was observed after 24 hours, and the prevascularized microtissues were integrated with the host vascular system after 4 weeks (Dissanayaka et al., 2015a).

##### 5.1.1.3 Synthetic scaffold based on gelatin methacrylate

Gelatin methacrylate (GelMA) is an inexpensive ultraviolet-cured synthetic material with excellent cell adhesion ability, physical and chemical adjustability and biodegradability and is beneficial to cell encapsulation, survival and proliferation. Khayat et al. encapsulated hDPSCs and HUVECs with GelMA and observed well-organized neovasculature formation (Khayat et al., 2017). However, the traditional ultraviolet irradiation crosslinking method produced oxygen free radicals, which destroyed endogenous DNA and led to tissue aging. Monteiro used an LED blue light source to irradiate GelMA containing encapsulated SCAPs/HUVECs, which not only avoided the harmful consequences of ultraviolet photopolymerization but also accelerated the formation of a wide range of vascular networks (Monteiro et al., 2018). Tian et al. incorporated human PL into a GelMA microsphere system to optimize the angiogenic activity and further modified it with laponite, which has a high drug delivery capacity, to improve the physical properties (Zhang Q. et al., 2021). One year later, this research team used GelMA sodium alginate core-shell microcapsules to solve the difficulty of collecting hydrogel microspheres and their easy aggregation (Liang X. et al., 2022). Both studies encapsulated hDPSCs and HUVECs to obtain highly prevascularized microtissues. In addition, the research team injected GelMA loaded with DPSCs or endothelial cells into transparent silicone tubes and crosslinked with ultraviolet to fabricate cell-laden microfibers. Two types of GelMA hydrogel microfibers loaded with HUVECs and DPSCs were respectively prepared and combined at a ratio of 2:1 to form microfiber aggregates (MAs). Compared with GelMA hydrogel blocks, the MAs have better mechanical properties, and their diameter of 400 μm was similar to that of microvessels, which enabled these materials to improve the survival rate of cells and induce the formation of more pulp-like tissue, blood vessels and odontoblast-like cells in an ectopic root model (Liang Q. et al., 2022).

##### 5.1.1.4 Synthetic scaffold based on other materials

The modification of other materials also achieved excellent results. Silva modified an injectable HA hydrogel with cellulose nanocrystals and added PL. Cellulose nanocrystals improved the mechanical properties and biological activities of HA while optimizing the release curve of angiogenic factors in PL (Silva et al., 2018). Lai took advantage of the potential of Si ions to promote angiogenesis by preparing cell blocks with alginate/fish gelatin gel rich in human DPSCs in the center and fish gelatin methacrylate rich in silicon ion implanted HUVECs in the periphery. The combination of alginate and gelatin optimized the physical and biological properties, and the modification of gelatin by methacrylate caused the gelatin to photopolymerize, improving the mechanical and physical properties. Finally, the synergistic cell block induced the formation of vascular structure and odontogenesis (Lai et al., 2021). Li combined VEGF with heparin and encapsulated VEGF in heparin-conjugated gelatin nanospheres. The nanospheres were fixed in nanofibers of injectable poly (l-lactic acid) microspheres. In summary, the VEGF binding domain of heparin prevented the denaturation and degradation of VEGF and controlled its slow release; the double-layer microsphere system simulated the ECM structure, effectively accommodated DPSCs to promote their proliferation, migration and differentiation, and cooperatively induced the formation of vascularized dental pulp tissue (Li et al., 2016).

#### 5.1.2 3D self-assembled microtissue constructs

The communication between cells and the cell‒ECM interaction in a 3D microtissue construct assembled by cells can mostly simulate the microenvironment *in vivo* and is not affected by materials. Dissanayaka constructed a scaffold-free microtissue sphere of hDPCs that was prevascularized by HUVECs in agarose 3D petri dishes and ultimately induced the regeneration of vascularized pulp-like tissue (Dissanayaka et al., 2014; 2015b). Itoh fabricated a 3D scaffold-free rod cell construct composed of DPSCs in a thermoresponsive hydrogel mold and formed dental pulp-like tissue with rich blood vessels in a model of tooth root ectopia. The part of the tissue in contact with the dentin wall differentiated into odontoblast-like cells, and human CD31^+^ ECs were found in the center of the tissue (Itoh et al., 2018). A previous study referred to Itoh’s method to fabricate 3D DPSCs constructs (cube structure), and then induced the formation of prevascularized endothelial differentiated cell constructs to achieve the regeneration of vascularized pulp tissue (Katata et al., 2021).

### 5.2 Top-down fabrication

Top-down fabrication uses different technologies, such as dissolution and sacrifice molding, 3D bioprinting, laser degradation, layer-by-layer fabrication and microfluidics (Miller et al., 2012; Song et al., 2018; Sharma et al., 2019), to prebuild vascular beds with specific geometric shapes. In the field of dental pulp regeneration, dissolution sacrificial molding is most commonly used. Sacrificial materials are added to the hydrogel to form hollow microchannels. ECs and pericytes or other related vascular cells are seeded into the inner wall of the channel to form microvessels. Other technologies in this field have yet to be developed.

Athirasala placed a prefabricated sacrificial agarose fiber in the root canal to create a microchannel through the full length of the root canal from the pulp chamber to the apical foramen. They placed GelMA hydrogel prepolymer loaded with odontocyte-like cells (OD21) in the root canal (around the microchannel) and aspirated the sacrificial fiber with a vacuum glass straw after photopolymerization. Then, endothelial colony forming cells were seeded into the microchannel, differentiated into monolayer ECs with vascular bud sprouting on the seventh day, and formed a full-length prevascularized pulp-like tissue structure (Athirasala et al., 2017).

Qi et al. utilized the template micromolding technique to create *in vitro* 3D engineered tissue constructs with inbuilt fluidic microchannels for dental pulp tissue regeneration. They first built PDMS molds that formed the fundamental part of the microchannel-on-a-chip and prepared agarose sacrificial template material. Then, parallel and different tapered templates were fabricated by injecting agarose solution into the PDMS molds using a glass capillary and gutta-percha points, respectively. The agarose template was assembled in the middle of the PDMS chip. Subsequently, the microchannel was formed by dispensing different concentrations of GelMA hydrogel precursor to cover the template, followed by photocrosslinking and removal of the template. Finally, HUVECs were seeded alone or with SCAPs in coculture into these microchannels. The coculture of HUVECs and SCAPs at a 1:1 ratio in 0.04 taper microchannels within 5% GelMA hydrogel scaffolds was the most conducive to stimulating sprouting angiogenesis. In addition, the preencapsulation of SCAPs in the GelMA hydrogel around the microchannel could further accelerate the process of sprouting (Qi et al., 2021)**.**

### 5.3 Future directions

Regarding construction strategies for prevascularization systems, we can focus on the following points in future research: further development of materials, including reducing the complexity of their preparation and improving their yield and properties. In addition, natural and synthetic materials are not completely oppositing and could potentially be combined. Zheng combined GelMA hydrogel microspheres with dental pulp derived growth factors to achieve multifunctional dental pulp regeneration by taking advantage of the former’s plasticity, biocompatibility and easy functionalization, and the latter’s ability to reduce the dental pulp ECM to induce DPSCs to differentiate in multiple directions (Zheng et al., 2023). Therefore, the proper combination of the two types of materials will be helpful to further optimize their performance based on the maximum simulation of the ECM and surpass the functional limitations of natural tissue. Although 3D self-assembled constructs can avoid the influence of materials and rely on the ECM secreted by cells to restore the microenvironment *in vivo*, challenges such as low production, high technical difficulty and high cost still need to be solved (Liang X. et al., 2022). The unique advantage of top-down fabrication is that it can personalize the root canals of different patients. However, at present, this strategy is rarely used in the field of pulp regeneration. Therefore, the development of other technologies can be considered while further studying dissolution sacrificial molding. In a word, the enrichment and optimization of the methodology can help the rapid development of prevascularization technology that can be initially applied in pulp regeneration and identify the most suitable construction strategy in the future.

## 6 Conclusion

At present, prevascularization techniques for dental pulp tissue engineering remain in their infancy. The cell sources of ECs and pericytes are limited and of low quality; the application and development of intercellular communication mechanisms and mediators are insufficient; the construction of prevascularization systems is based mainly on dissolution and sacrifice molding technology, and research on other technologies remains insufficient. In fact, expanding the range of cell sources and enriching and optimizing the acquisition methods could significantly improve the output and quality of ECs and pericytes, providing strong support for laboratory research and subsequent clinical transformation; an improved understanding and application of intercellular communication, such as taking signaling pathways as therapeutic targets or developing potential signaling mediators, including natural signal molecular storage and EVs, could further accelerate the process of vasculogenesis and angiogenesis in the coculture of ECs and pericytes; cell‒ECM interaction mainly occurs in the prevascularized systems after the implantation of EC-pericyte coculture (dental pulp engineered tissue). Therefore, suitable construction strategies for prevascularization systems, including bottom-up and top-down fabrication, would be conducive to the efficient prevascularization of engineered tissue before implantation and to rapid anastomosis with the host vessel after implantation. The combination of these three factors would ultimately achieve prevascularization in pulp regeneration. This manuscript provides novel ideas for the extensive application and follow-up development of prevascularization techniques for dental pulp regeneration and could thus be used as a reference for future research.

## Figures and Tables

**Table:** 

**3D**	Three-dimensional
**ALK1**	Activin receptor-like kinase-1
**ALK5**	Activin receptor-like kinase-5
**ANGPT1/TIE2**	Angiopoietin-1/TEK receptor tyrosine kinase
**ASCs**	Adult stem cells
**BCL2**	BCL2 apoptosis regulator
**Cell‒ECM interaction**	Interaction between cells and the extracellular matrix
**CXCL12**	C-X-C motif chemokine ligand 12
**CXCR4C**	C-X-C motif chemokine receptor 4
**DPSCs**	Dental pulp stem cells
**DSCs**	Dental stem cells
**ECM**	Extracellular matrix
**ECs**	Endothelial cells
**EFNB2/EPHB4**	Ephrin B2/EPH receptor B4
**ETV2**	ETS variant transcription factor 2
**EVs**	Extracellular vesicles
**FGF**	Fibroblast growth factor
**FGF2**	Basic fibroblast growth factor
**GelMA**	Gelatin methacrylate
**GSK-3b**	Glycogen synthase kinase 3 β
**hDPCs**	Human dental pulp cells
**hDPSCs**	Human dental pulp stem cells
**HIF-1α**	Hypoxia inducible factor 1 subunit alpha
**HUVECs**	Human umbilical vein endothelial cells
**iPSCs**	Induced pluripotent stem cells
**MSCs**	Mesenchymal stem cells
**PDGF-BB**	Platelet-derived growth factor BB
**PDGFRB**	Platelet-derived growth factor receptor
**PL**	Platelet lysate
**SCAPs**	Stem cells from apical papilla
**SHED-Exo**	Exosomes derived from SHEDs
**SHEDs**	Stem cells of human deciduous exfoliated teeth
**SMA**	Smooth muscle α-actin
**SMCs**	Smooth muscle cells
**T-DPSCs**	DPSCs treated with TGF-β
**TGF-β**	Transforming growth factor beta
**VE-cadherin**	Vascular endothelial cadherin
**VEGF**	Vascular endothelial growth factor
**VEGFR2**	Vascular endothelial growth factor receptor 2
**VWF**	Von Willebrand factor
